# A Semi-Automated and Unbiased Microglia Morphology Analysis Following Mild Traumatic Brain Injury in Rats

**DOI:** 10.3390/ijms26178149

**Published:** 2025-08-22

**Authors:** Luke Sumberg, Rina Berman, Antoni Pazgier, Joaquin Torres, Jennifer Qiu, Bodhi Tran, Shannen Greene, Rose Atwood, Martin Boese, Kwang Choi

**Affiliations:** 1 Center for the Study of Traumatic Stress, Uniformed Services University, Bethesda, MD 20814, USA; luke.sumberg.ctr@usuhs.edu (L.S.); rina.berman.ctr@usuhs.edu (R.B.); 2 Department of Computer Science and Electrical Engineering, University of Maryland, Baltimore County, Baltimore, MD 21250, USA; antonip1@umbc.edu; 3 Department of Biological Sciences, University of Pittsburgh, Pittsburgh, PA 15260, USA; joaquintorr007@gmail.com; 4 Department of Biology, University of Maryland, College Park, MD 20742, USA; jenniferqiu265@gmail.com; 5 Bethesda Chevy Chase High School, Bethesda, MD 20815, USA; bodhitran1@gmail.com; 6 Winston Churchill High School, Potomac, MD 20854, USA; shannenrosegreene@gmail.com; 7 Department of Psychology, University of Colorado, Boulder, CO 80309, USA; roseatwood05@gmail.com; 8 Daniel K. Inouye Graduate School of Nursing, Uniformed Services University, Bethesda, MD 20814, USA; martin.boese@usuhs.edu; 9 Walter Reed National Military Medical Center, Bethesda, MD 20814, USA; 10 Program in Neuroscience, Uniformed Services University, Bethesda, MD 20814, USA; 11 Department of Psychiatry, Uniformed Services University, 4301 Jones Bridge Rd, Bethesda, MD 20814, USA

**Keywords:** mild traumatic brain injury, microglia morphology, neuroinflammation, sprague-dawley rats, cortical injury

## Abstract

Mild traumatic brain injury (mTBI) affects over 40 million people every year. One of its features includes the activation of microglia, the resident immune cells of the brain. Microglia assume different morphological states depending on their level of activation, such as surveilling ramified and activated hypertrophic, ameboid, and rod-like microglia. These states can be distinguished by multiple features, including the shape, span, and branching of microglia. Male Sprague–Dawley rats sustained mTBI using the Closed-Head Impact Model of Engineered Rotational Acceleration (CHIMERA) (3 times, 1.5 J per impact) or sham treatment. Four days after the injury, brains were collected and stained for microglia using the ionized calcium-binding adapter molecule-1 (Iba-1) antibody. Cortical injury sites were identified in a subset of CHIMERA animals. Using the MicrogliaMorphology ImageJ plugin and the MicrogliaMorphologyR package, 27 morphological features were quantified from individual microglia, and k-means clustering was used to classify microglia as ramified, rod-like, ameboid, and hypertrophic states. The CHIMERA injury altered microglia morphology features, which contributed to increased hypertrophic (activated) and decreased ramified (inactive) microglia compared to the sham controls. Combined with the clinically relevant mTBI paradigm and semi-automated/unbiased approach, the current findings may contribute to microglia morphology classification.

## 1. Introduction

Mild traumatic brain injury (mTBI) is a leading public health concern. The majority of TBI cases (80%) are categorized as mild [1]. While most patients with mTBI recover normally from their injury, a small subset of the population (10–30%) has long-term consequences [2,3,4], including cognitive disturbances, physical imbalances, and emotional sequelae [5]. These problems can stem from neuroinflammatory processes that happen days or months after the injury. One such process is the activation of microglia, the neuroimmune cells in the brain that play a role in mediating injury and disease [6]. However, microglia actions can be neuroprotective or neurotoxic depending on various factors, including the severity and timing of injury, inflammation, and the condition of injured cells [7].

Additionally, microglia undergo morphology changes in response to injury and stress. In homeostatic conditions, microglia assume a ramified state with long, highly branched processes. Under injury or other neurodegenerative stimuli, microglia retract their processes and enlarge their cell bodies; this is referred to as an ameboid state [8]. Ameboid microglia are considered to be fully activated, having phagocytic properties to remove injured cells and debris. There are also intermediate states that exhibit characteristics of both inactive and activated microglia. One of these transitional states is hypertrophic microglia, also named “activated” or “primed” [9] microglia, which are generally characterized by having retracted, thickened processes [10,11]. Microglia may also assume a rod-like state, an injury-associated phenotype with elongated, narrowed somas and polarized processes [12,13]. In rats sustaining fluid percussion TBI, rod-like cells formed long trains in the injured cortex—a phenotype not seen in sham controls [14]. Thus, the analysis of microglia morphology can be useful for assessing microglial function at a specific time window to infer the neuroinflammatory stages of the brain after TBI.

However, the differences between morphological states can be subtle and heterogeneous, making it a labor-intensive and time-consuming task to analyze tens of thousands of microglia. Especially in manual analysis, the criteria for determining microglial morphology may differ between laboratories and investigators [9], and may fail to account for heterogeneity in microglial characteristics across brain regions, species, age, sex, and injury or disease states [13,15]. Accordingly, implicit bias may influence researchers to disproportionately trace and include microglia whose morphology supports their hypothesis [9]. Automated techniques of measuring individual morphological features can increase the efficiency of morphology analyses while providing insight into their morphological characteristics. For example, skeleton analysis simplifies the structure of microglia into a skeletal framework to extract and quantify morphological features pertaining to branch length and complexity, and it can be applied to numerous microglia simultaneously [16]. Additionally, fractal analysis quantifies the size, shape, and span of individual microglia through fractal dimension analysis [8]. Thus, these automated techniques reduce the biases introduced by manually selecting and tracing microglia, and drastically increase the amount of microglia that can be analyzed in a short period of time. Moreover, current advances in machine learning algorithms in imaging data analysis can enhance the efficiency and sensitivity of microglia morphology analysis.

Several studies have found that microglial activation is increased at the injury site of an mTBI. Typically, activated microglia, which are described as having intense staining and shorter, thicker processes, are quantified and found to be increased after mTBI. For example, activated microglia, marked by dark Iba-1 staining and thickened processes, were upregulated in the injured cortex in a weight drop model in rats [17]. Similarly, the levels of activated microglia marked by intense CD11b staining and shorter, thicker processes were increased in the medial cortex directly underneath an impactor strike in mice [18]. However, this binary approach and lack of feature quantification led to the exclusion of other morphological subtypes. A study using medial fluid percussion (MFP) injury in mice examined endpoints per cell, span ratio, and fractal dimension in the injured cortex, the primary somatosensory barrel cortex (S1BF), and a remote site over multiple time points [19]. Microglia were classified into ramified, ramified and hyper-complex, de-ramified, and de-ramified and rod-like. Generally, the impact site had de-ramified cells, and the S1BF had de-ramified and rod-like cells, consistent with the previous literature [20]. In contrast, the remote site had ramified and hyper-ramified cells [19]. This approach could be further improved by incorporating additional morphological features to enhance sensitivity.

Most preclinical mTBI studies utilize an open-head injury, which requires a craniotomy to administer cortical injury directly to the brain. Similarly, several studies use a head restraint in a stereotaxic frame. Neither of these scenarios models human mTBI patterns through blunt force trauma or a blast injury to an intact, unrestrained head. Thus, the current study used rat brains collected from the previous study that utilized the Closed-Head Impact Model of Engineered Rotational Acceleration (CHIMERA), which closely simulates human TBI as previously reported [21,22]. This technique delivers a mechanical impact to an unrestrained head, allowing it to rotate freely. The CHIMERA model can be used in different species, including ferrets [23], mice [21,22,24,25,26], and rats [27,28,29]. However, previous studies have not adopted the semi-automated and unbiased approach of determining microglia morphology following CHIMERA injury in rats.

The current study utilized a semi-automated and unbiased microglia morphology analysis pipeline developed in a recent study [30]. This approach extracts individual microglia cells from Iba-1-stained photomicroscopic images and calculates 27 morphological features based on the skeleton and FracLac analyses. This information is combined and analyzed using a principal component analysis (PCA) and an unsupervised k-means clustering to assign each microglia to one of the four major clusters: ramified, hypertrophic, rod-like, and ameboid. The main goal of this study was to validate this analysis approach using mTBI samples in rats. We hypothesized that CHIMERA injury may increase activated forms of microglia and reduce the inactive microglia in the cortical injury sites of rats.

## 2. Results

### 2.1. Dimensionality Reduction Analyses

**Figure 1:**
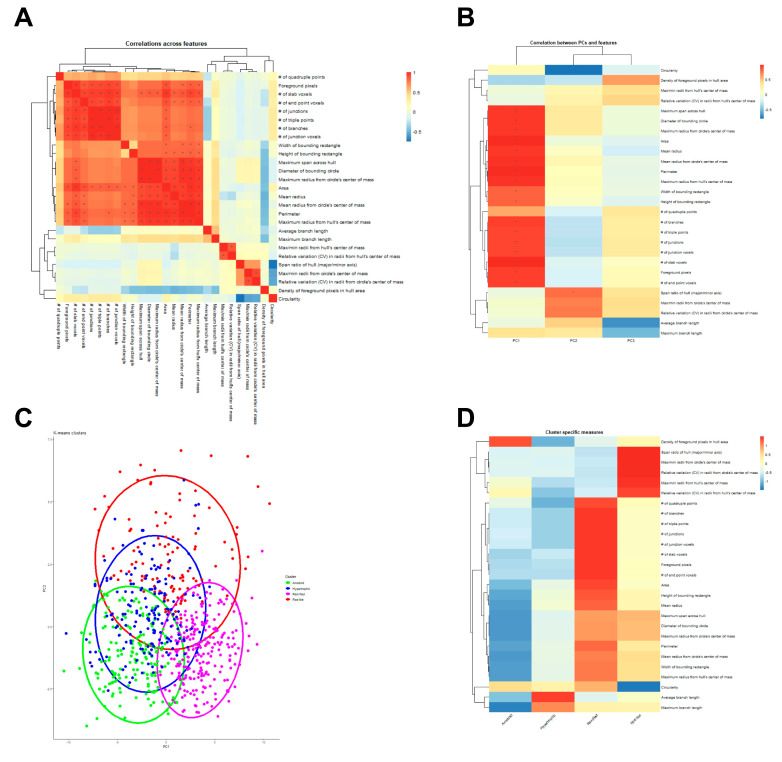
Analyses of microglia morphology features (**A**) Heatmap of correlations between individual morphology features. Spearman’s correlation analysis was used to calculate the correlation between each pair of morphology features. Significance was determined by * |R| ≥ 0.75, *p* < 0.05. (**B**) Heatmap of correlations between principal components (PCs) and morphology features. The correlations between the 27 morphological features and the first three PCs are shown. Significance was determined by * |R| ≥ 0.75, *p* < 0.05. (**C**) A Venn diagram of k-means clustering (k = 4) plotted along the first two PCs. Individual microglia were assigned to one of the four clusters, each representing one morphologic state. (**D**) The morphological states represented by the four clusters in (**C**) were identified based on their features, visualized in a cluster-specific measures heatmap. The four clusters were identified as ameboid, hypertrophic, ramified, and rod-like, respectively.

Spearman’s correlation was used to produce a correlation matrix demonstrating correlations between 27 morphology features in the heatmap (Figure 1A). As expected, morphology features related to territory span and branching complexity were highly interrelated, as were features related to shape heterogeneity/oblongness (Figure 1A). The PCA on 27 morphology features indicated that the first three principal components (PC1, PC2, and PC3) captured the majority of the variability in the dataset. PC1 was associated with low area and territory span as well as low branching complexity, PC2 was associated with low shape heterogeneity, and PC3 was associated with high average and maximum branch length (Figure 1B). K-means cluster memberships for each microglia were assigned using scaled values of the first three PCs, and microglia clusters were plotted according to each cell’s values on the first two PCs. A Venn diagram with 4 clusters indicates the intersect between those clusters, including rod-like (red), ameboid (green), hypertrophic (blue), and ramified (magenta) in Figure 1C. There was significant overlap between the clusters, especially between hypertrophic and ameboid clusters. This suggests that the current classification method using these morphology features requires further improvement and stratification in data analysis. Cluster-specific morphology features were visualized using a heatmap with hierarchical clustering (Figure 1D). For example, the ameboid type was associated with a high density of foreground pixels in the hull area and low territory span, while the hypertrophic type was associated with average territory span and high average and maximum branch length. In contrast, the ramified type was associated with higher territory span and branching complexity, while the rod-like type was associated with high shape heterogeneity and oblongness (e.g., high variation in max/min radii size, span across hull and bounding circle).

### 2.2. Morphological Feature Comparison

The values of individual microglia morphology features were compared between the sham and CHIMERA groups. Features were stratified similarly to the previous study [30], and the results are presented in Table 1. For example, microglia in the sham group had higher levels of foreground pixels, number of junctions and junction voxels, and number of triple points compared to the CHIMERA group. Notably, other features such as number of branches and number of slab voxels had near-significant increases in the sham group (0.05 < *p* < 0.1).

### 2.3. Microglia Density and Morphology

**Figure 2:**
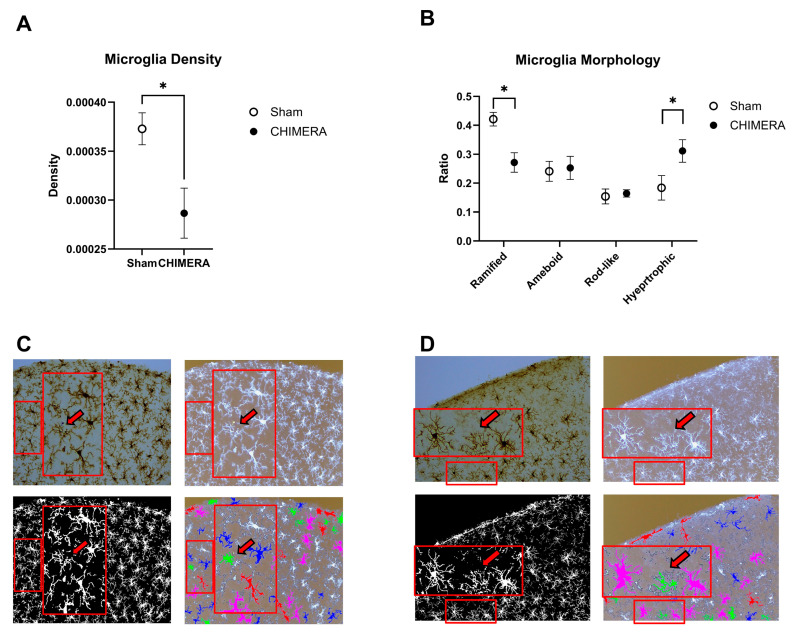
Comparison of microglia density and morphology between the sham and CHIMERA groups. (**A**) Microglia density is decreased in the CHIMERA group compared to the sham controls. Density was calculated by dividing the number of microglia in the brain area. (**B**) The CHIMERA injury reduced the ratio of ramified (inactive) and increased hypertrophic (activated) microglia compared to the sham controls. (**C**) Representative images (DAB, inverted, binary, and ColorByCluster on the inverted) from the sham control brain (a clock-wise direction from the top left). Unlabeled microglia (shown in white) were excluded from the morphology analysis based on the cell size cutoff (e.g., <150 µm^2^ or >1000 µm^2^). A larger rectangle is a magnified image of a smaller rectangle with the arrow indicating a false-positive ameboid type. (**D**) Representative images (DAB, inverted, binary, and ColorByCluster on the inverted) from the CHIMERA brain. Unlabeled microglia (shown in white) were excluded from the morphology analysis based on the cell size cutoff (e.g., <150 µm^2^ or >1000 µm^2^). A larger rectangle is a magnified image of a smaller rectangle, with the arrow showing a false-positive ameboid type. * *p* < 0.05. Data presented as mean ± SEM. Scale bar: 100 µm.

Density of microglia in the cortical injury sites was compared between the CHIMERA and sham groups. The unpaired *t*-test indicated that CHIMERA injury reduced microglial density in the cortical injury sites compared to the sham group (t = 2.846, df = 11.88, *p* = 0.015), as shown in Figure 2A. Using 27 morphology feature data, k-means clustering was performed to determine four major morphology types in microglia, such as ramified, hypertrophic, rod-like, and ameboid. The ratios of microglia belonging to four different clusters were compared between the sham and CHIMERA groups (Figure 2B). A two-way ANOVA on cluster ratios indicated a significant main effect of microglia clusters (F (3, 52) = 13.23, *p* < 0.0001) and interaction between injury and morphology (F (3, 52) = 6.304, *p* = 0.001), but not CHIMERA. Post hoc analysis using the FDR method of Benjamini–Hochberg revealed significant differences in ramified (q-value: 0.016) and hypertrophic (q-value: 0.007) clusters between the sham and CHIMERA groups. The current CHIMERA paradigm increased hypertrophic (activated) and reduced ramified (inactive) microglia in the cortical injury sites as compared to the sham control. However, other active forms, such as rod-like and ameboid, were not significantly different between the groups.

To verify the accuracy of microglia morphology classification using k-means clustering, the ColorByCluster function was used to color-code individual cells into four clusters. Four different images from the same brain section that were included in this analysis are shown in Figure 2C (sham) and 2D (CHIMERA). The DAB-stained image (upper left) and the inverted black and white image (upper right) clearly show detailed cell body and branch information, while the binary black and white image (lower left) does not. However, it is necessary to convert the images to binary format to perform skeleton and fractal analysis on individual microglia. The ColorByCluster image (lower right) mainly depicts the shape and size of individual microglia without detailed cell body information. This can lead to some issues with the classification of certain cells. For example, there were false-positive ameboid cells based on this current clustering algorithm, as shown inside the images (Figure 2C,D). Those cells (shown with the red arrows) do not exhibit clear cell bodies in DAB-stained and inverted images; therefore, they are more likely fragments or cross-sections of other types of microglia rather than true ameboid cells.

## 3. Discussion

Following a repeated CHIMERA injury in a single session, a subgroup of the animals (approximately 25%) showed clear signs of mild cortical injury at PID-4. Thus, microglia density and detailed microglia morphology were examined using a recently developed semi-automated and unbiased approach [30]. The presence of an increased ratio of activated hypertrophic microglia and decreased inactive ramified microglia in the cortical injury sites of the CHIMERA group validates the utility of this analysis method in a rodent model of mTBI.

These changes lead to shifts in the distribution of microglia morphology states. Sham control brains had significantly more ramified cells compared to other morphology states. In contrast, all four morphology states were relatively evenly distributed in the CHIMERA brains, because the increase in the hypertrophic state and decrease in the ramified state were roughly equal. We did not observe changes in the ameboid shape between the groups, which may be due to the mild nature of the current injury paradigm, as hypertrophic cells are recruited with more mild brain injuries, while ameboid cells are activated with more severe brain injuries, as described previously [31]. In addition to the morphology, microglia density was also reduced following CHIMERA injury, suggesting a relatively mild injury rather than a severe injury in this study. A previous study also reported that the controlled cortical impact (CCI) model decreased the number of rod-like microglia in galectin-3 gene knock-out mice [32]. It has been suggested that rod-like microglia are another form of activated microglia that can be found in the ageing, TBI, and other diseased conditions in the brain [33]. Although staining microglia using Iba-1 antibodies cannot distinguish between microglia and monocyte-macrophages in the brain, macrophages are generally more round and circular in shape compared to microglia, and we have not found many shapes resembling macrophages in our study. Thus, the ameboid-like cells identified in this study are unlikely to be derived from macrophages.

Differences in microglia morphological features that aligned with these changes in cluster membership were detected. Microglia in the sham group had higher scores in most metrics of branching complexity derived from the skeleton analysis and foreground pixels related to area and territory span. In general, microglia in the sham group occupied more space and exhibited more intricate branching patterns, in alignment with the higher representation of ramified microglia.

Using 27 morphological features may enable investigators to perform more granular analysis, which can be missing in conventional analyses that use a smaller number of morphology features. Hypertrophic cells, which are an intermediate stage that can be difficult to define, present an important example. Ramified cells are widely recognized as having a high complexity of branching and extended processes. However, unsupervised k-means clustering analysis reveals that hypertrophic cells have larger average branch length, ostensibly because their processes have fewer arborizations and remain relatively uninterrupted rather than diverging to form many smaller branches. Thus, studies with fewer metrics of branching length and complexity may have missed these subtle differences in microglia morphology. Additionally, hypertrophic cells lacked elevated foreground pixel density, which represents the branch/soma thickness characteristics of this morphologic state, but they were still easily distinguishable due to average territory span and high average and maximum branch length. Thus, the presence of many features allows for the detection of morphology with high sensitivity.

There are several technical limitations when using this analysis approach. For instance, hypertrophic cells are an intermediary state and share characteristics with other clusters, such as foreground pixel density in the hull area (soma/branch thickness) with ameboid cells, and some shape heterogeneity with rod-like cells. Therefore, these cells may be poorly defined. In addition, there were other issues associated with determining true ameboid cells, such as branches without the cell body and cross-sections of non-ameboid cells in a two-dimensional plane due to tissue sectioning. Thus, we compared different ranges of size exclusion criteria (e.g., 50, 100, 150 µm^2^) as well as different image thresholding at the image pre-processing step to optimize the analysis condition (e.g., maintain sufficient cell body and branch information while minimizing the condition that two cells appeared to be connected in a two-dimensional plane). Taken together, fine-tuning of multiple parameters at the pre-analysis stages may have a significant impact on the outcome of the analysis.

These technical limitations are important to consider despite using 27 morphological features for clustering on microglia morphometrics, especially in different severities of TBI. For example, the current approach can be effective in moderate-to-severe TBI cases when there is a clearer distinction between activated and inactive microglia at local injury sites [31]. However, in the case of mTBI or blast injury without direct impact to the head, the clustering of microglia morphology can be challenging, even with those rich morphological features, due to the inherent heterogeneity and diversity of microglia shapes. A recent review article discussed the importance of accurate visualization and microglia morphology determination using cerebral biopsy samples from TBI patients to avoid potential misinterpretation [34]. Given the possibility of collecting fresh brain tissue from living patients suffering TBI [35], optimizing microglia morphology analysis can provide precise and accurate information on neuroinflammation and secondary injuries in the brain. In animal studies, microglia in mice may have heterogeneous cell body shapes and sizes, which hampers the distinction between ramified and hypertrophic microglia [36]. Therefore, additional analysis and functional studies are needed to validate and complement microglia morphology analysis across various species.

It is important to note that the current CHIMERA paradigm produced cortical injury in a subset of animals (approximately 25%). The precise location of the cortical injury sites was variable (2.76 to 4.2 mm medial-lateral to Bregma, 13.2 to 11.52 mm interaural), which may have been due to repeated closed head impacts with a relatively large-sized piston (10 mm in diameter) aimed at the Bregma area of the head. Another potential reason that not every CHIMERA animal may show cortical injury is the sampling of brain sections for analysis. One in every 12 sections was selected for the immunohistochemistry experiment, covering areas with approximately a 480 μm interval between the sections. Thus, cortical injury sites in some animals may have been missed due to this sampling procedure. On the contrary, previous rodent studies, using more severe CHIMERA paradigms (repeated injuries over multiple days), found multiple behavioral deficits (e.g., righting reflex, neurological severity, and impulsivity) and brain pathology (e.g., axonal injury and glial activation in multiple brain regions) [22,28,29]. The main goal of the current analysis was to validate and optimize the morphology analysis condition using a subset of mTBI samples showing cortical injury signs based on global Iba-1 immunostaining density and intensity.

The current study investigated the effects of mTBI on microglia activation, which is the most common form of TBI in the military by incidence [1]. However, mTBI is difficult to quantify because the injury effects are often subtle and transient, with only a small subset of the population having long-term consequences [2,3,4]. Given that heterogeneity in injury pathology is a key characteristic of mTBI, the fact that only a fraction of rats in our study demonstrated clear signs of cortical injury is clinically relevant. The current study achieved rates of visible cortical injury similar to analogous phenotypes in human mTBI. Patients with mTBI have wide variability in their pathophysiology, with the majority having no intracranial abnormalities [37]. However, a minority show radiological findings with otherwise mild symptomology- a classification referred to as “complicated mTBI”, which according to some estimates encompasses 5–30% of mTBI patients [37,38]. A review of 3032 computed tomography (CT) scans in emergency patients with suspected TBI revealed that 16% of mTBI patients showed intracranial abnormalities such as hemorrhage, hematoma, and contusion [39]. Similarly, 32% of mTBI patients showed abnormalities such as contusion and gliosis on magnetic resonance imaging (MRI) [40]. Thus, the relative scarcity of visual abnormalities in the cortex after CHIMERA injury in our rodent study is consistent with the clinical literature on mTBI.

Another possible reason explaining our observance of relatively few visible injury sites is the timing of data collection. This data was collected from rats whose brains were obtained on day four in our previous studies [27]. This time point was chosen because microglia appear to become activated around 4 days after TBI [41,42], and it is incidentally important for synaptic analysis [43]. However, microglial activation and morphology can change dynamically over time. The primary peak of microglial activation after TBI is noted to be at around 7 days [44]. In addition, microglia in mice sustaining an MFP had dynamic changes in size, complexity, and span ratio over 1, 7, and 28 days post-injury, and their morphology changed accordingly over time [19]. Due to the subjectivity of determining a consistent region of interest (ROI) following mTBI, we compared the analysis results with or without an ROI drawn on the cortical injury sites. Thus, the overall areas of the brain tissue analyzed between the CHIMERA and sham groups are comparable. Overall results between these analyses were similar, indicating increased hypertrophic (activated) microglia and reduced ramified (inactive) microglia on the cortical injury sites of the CHIMERA animals as compared to the matched areas of the sham animals.

Determining the accuracy of microglia morphology, especially ameboid-like cells, can be challenging due to the heterogeneity of sizes and shapes of microglia. However, this can be improved by the fine-tuning of parameters, including image thresholding, cell size cutoff range, and classification algorithms. Specifically, more advanced machine learning algorithms can be used to improve the classification accuracy of microglia morphology. The quality of data analysis could be improved by increasing statistical power (number of animals and cells), including the detection of multiple brain regions of the same animals, the double labeling of microglia with specific antibodies, and the identification of additional morphology features for individual cells. Using immunofluorescent antibodies specific to different activation states of microglia, combined with nuclear counterstaining, may further enhance the classification of morphology by reducing false positives (e.g., branches without a cell body or cross-section of microglia in a two-dimensional plane due to brain tissue sectioning). Moreover, we performed k-means clustering with larger numbers of clusters (k = 5–8) and found additional clusters with new morphology characteristics that require further investigation in future studies.

## 4. Materials and Methods

### 4.1. Animals

Brain tissue samples analyzed in this study were collected from the previous study that reported behavioral effects of CHIMERA and ketamine in rats [27]. Adult male Sprague–Dawley rats (9 weeks old upon arrival) were housed individually in clear Plexiglass cages in a climate-controlled environment. Animals were maintained on a reversed 12-h light/dark cycle (lights off at 0600 and on at 1800) with food and water available ad libitum. A jugular vein catheter (polyurethane; Instech, Plymouth Meeting, PA, USA) was surgically implanted under isoflurane anesthesia at the Envigo Laboratories (Indianapolis, IN, USA). Catheters were flushed twice weekly to maintain catheter patency and locked with 0.1 mL sterile heparin/glycerol solution (1:1 dilution). 

### 4.2. CHIMERA

Each animal received either mTBI (CHIMERA) or sham treatment in a single session [27]. Briefly, animals were anesthetized with isoflurane (4% for induction and 3% for maintenance) mixed with 100% oxygen. Each animal was strapped in a dorsal position on the CHIMERA device, and the head was positioned over the crosshair on an aluminum plate, aligning the impact piston (10 mm diameter) approximately above the Bregma area [21,28]. A hole in the plate allowed a 200 g piston to impact the head (1.5 Joules, 5.5 m/s velocity). Animals in the CHIMERA group received three impacts to the head in a single session, with a 5–10 s interval between impacts, whereas animals in the sham group received the identical treatment without actual impact to the head. All animals were given the choice to drink diluted acetaminophen (1 mg/mL) water for one day in addition to regular tap water.

### 4.3. Iba-1 Immunohistochemistry

These brains were collected at post-injury day 4 (PID-4) to target the timeline of the early stage of microglial activation in the brain after TBI as previously reported [41,42,44]. Formalin-fixed and cryoprotected brain tissue samples were cut using a freezing microtome, and Iba-1 immunohistochemistry was performed [45]. Briefly, free-floating brain sections (40 µm) were treated with 0.3% hydrogen peroxide solution for 30 min and with blocking buffer containing 10% normal goat serum (Vector Laboratories, Burlingame, CA, USA) and 0.02% bovine serum albumin (Fisher Scientific, Waltham, MA, USA, Fraction V) for one hour. Brain sections were incubated in a primary antibody solution (1:1000) of ionized calcium-binding adaptor molecule 1 (Iba-1) (Fujifilm Wako, Osaka, Japan, REF: 019-19741) at 4 °C overnight. The next day, sections were incubated with a secondary antibody solution (biotin-SP-conjugated goat anti-rabbit, Jackson Immunoresearch, West Grove, PA, USA) in a 1:300 dilution for one hour. Sections were then treated with the ABC solution (Vectastain ABC Peroxidase kit, Vector Laboratories, Burlingame, CA, USA) and stained with a 3,3′-diaminobenzidine (DAB) peroxidase substrate kit (with nickel) and 3,3′-diaminobenzidine staining solution (Vector Laboratories, Burlingame, CA, USA). Sections were mounted on glass slides and dried overnight. As a final step, sections were dehydrated using different concentrations of ethanol (75, 90, 95, and 100%), cleared in xylene, and then cover-slipped using Permount mounting medium (Fisher Chemical, Waltham, MA, USA).

### 4.4. MicrogliaMorphology

The MicrogliaMorphology method, an open-source and semi-automated ImageJ (ver. 2.14.0) macro and R (ver. 4.2.3) package, was used to analyze microglia morphology as previously described [30]. This MicrogliaMorphology ImageJ macro calculates 27 morphological features from individual microglia, as shown in Table 1. The associated R package, MicrogliaMorphologyR, (last accessed on July 2025) was used to process those 27 features utilizing built-in data quality control, dimension reduction, classification, and statistical analysis functions. The MicrogliaMorphology ImageJ macro and MicrogliaMorphologyR package are available from the GitHub (last accessed on July 2025) repository: https://github.com/ciernialab/MicrogliaMorphology; https://github.com/ciernialab/MicrogliaMorphologyR accessed on 18 August 2025.

#### 4.4.1. Cortical Injury Sites

**Figure 3:**
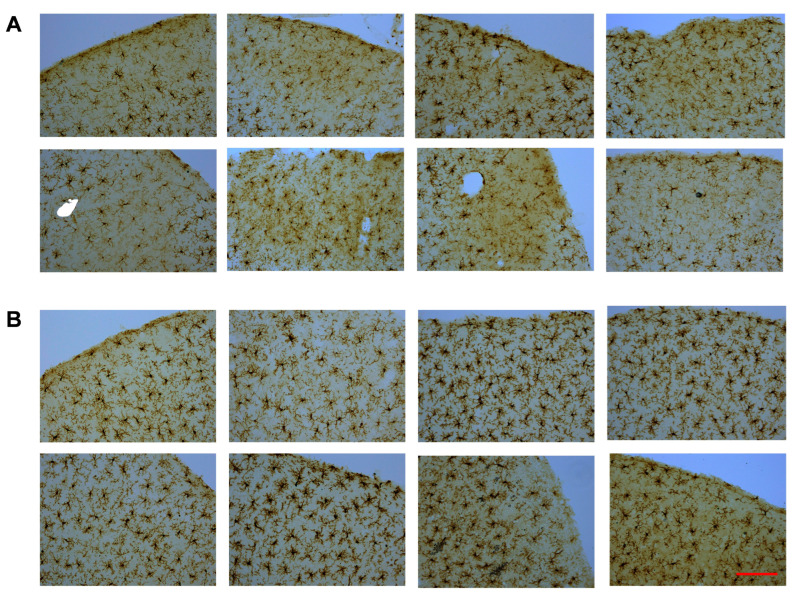
Iba-1-stained microglia images from CHIMERA and sham groups. Cortical injury sites from CHIMERA animals (**A**) and matched cortical regions from sham animals (**B**). Magnification (20×). Scale bar: 100 µm.

Iba-1-stained cerebral cortex sections were used to acquire microglia morphology images using a Motic BA310 Digital LED Compound Microscope at 20× magnification. Pre-determined criteria for mild injury signs without any brain tissue loss, such as reduced microglia staining density and darker background staining, were used to identify injury sites in CHIMERA animals. Each cortical section was scored as having either a probable injury site, a barely visible injury site, or no injury site. If a minimum of three brain sections from the same animal showed cortical injury signs, that animal was included for analysis to rule out false-positive findings. It is important to note that the current CHIMERA paradigm did not produce any obvious cortical brain tissue loss, which is a sign of mild closed-head injury (Figure 3A).

For validation, sham control brains were scanned for potential cortical injury using the same criteria mentioned above, and no signs of mild injury were detected in the sham brains. Each CHIMERA brain section with an injury site was matched to a sham brain section with approximately the same location using the Rat Brain Atlas as a guideline [46], as shown in Figure 3B. Microscopic evaluation of cortical injury sites around the Bregma area indicated that a subgroup of CHIMERA animals (approx. 25%) showed clear signs of cortical injury based on the criteria described above. Thus, only that subgroup of CHIMERA animals and matched sham animals (*n* = 8 per group) were used in the current analysis.

#### 4.4.2. Microglia Morphology Features

DAB-stained Iba-1 images (20×) were opened in ImageJ software (ver. 2.14.0). Because the MicrogliaMorphology plugin is optimized for fluorescent-labeled cells in a dark background, the DAB chromogen-stained images were inverted to enhance compatibility with this ImageJ plugin. The inverted images were subjected to the Threshold Check function in the MicrogliaMorphology BioVoxxel Toolbox plugin, which provides sixteen auto thresholding options and nine auto local thresholding options. Each threshold uses a different algorithm to define pixels as foreground (i.e., microglia) and background. The process of selecting an optimum threshold is crucial for maintaining a balance between preserving sufficient branch/process information in individual microglia (e.g., signal) and minimizing overlapping cells (e.g., noise).

Next, the cell size filtering step was implemented to exclude cells that were too small or too large before the analysis. For the lower boundary, ameboid-type cells (typically 100 µm^2^ or larger) were used as a reference point as previously reported [9]. For the upper boundary, 1000 µm^2^ was selected, as microglia larger than this size may result from two overlapping cells connected by their branches/processes. Using a size cutoff (150–1000 µm^2^), each microglia sample was extracted from the entire image. These individual cells were processed using skeleton analysis, which utilizes the Skeletonize and AnalyzeSkeleton plugins to calculate nine morphological features related to the length and complexity of branches for individual microglia. Individual cells were also processed in the separate FracLac Suite plugin, which yields one text file with information on 18 morphological features pertaining to the size, shape, and territory span of individual microglia.

#### 4.4.3. Microglia Morphology Clusters

**Figure 4:**
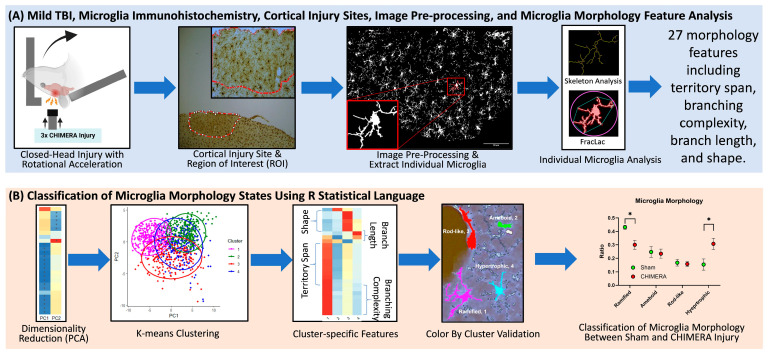
Overall study design and data analysis workflow. (**A**) CHIMERA injury and microglia morphology data acquisition. Brain tissue of rats sustaining a within-session repeated head impacts were collected, stained for microglia using Iba-1 immunohistochemistry, and cortical injury sites were identified at 4× and 10× magnification. Images (20×) were inverted, then processed and analyzed for 27 morphological features using the previous protocol [30]. (**B**) Data analysis. Microglia data were analyzed with principal component analysis (PCA), k-means clustering, cluster-specific features, and validated using the ColorByCluster function [30]. Morphological features and clusters were compared between the sham and CHIMERA groups, * *p* < 0.05.

The MicrogliaMorphology R package was used to perform data quality control analyses, multidimensional data reduction, unsupervised machine learning (k-means clustering), and data visualization. The results from the skeleton analysis and FracLac were imported and merged into a single data table, containing information on 27 morphological features for individual microglia. Data were log transformed and analyzed using a principal component analysis (PCA), a technique for dimensionality reduction to simplify the dataset and highlight the combinations of features that contribute most to variability in the data. Data were plotted along the first two principal components (PCs), and k-means clustering, an unsupervised machine learning algorithm, was used to classify cluster membership into four groups: (1) ramified, inactive microglia which have extended and thin branch processes; (2) hypertrophic, activated microglia with thicker and shorter processes; (3) rod-like, an injury-associated phenotype with an elongated shape; and (4) ameboid, the most activated microglial state with a round shape and few to no processes. A heatmap with hierarchical clustering of individual morphology features was generated to identify the pattern of morphologic state represented by each cluster. The ColorByCluster function in R was used to assign different colors to different clusters, and color-coded microglia were visualized. Microglia in white (unlabeled cells) were excluded because they fell outside the cell size cutoff set earlier or were outside of the analyzed region. Once the morphology clusters were validated, data were plotted as the percentage of microglia belonging to each morphological state in each animal. These data were averaged and compared between the sham and CHIMERA groups. The overall analysis pipeline is shown in Figure 4.

### 4.5. Statistical Analysis

Microglia density data were analyzed using an unpaired *t*-test with Welch’s correction. Microglia morphology data were analyzed using a two-way ANOVA with CHIMERA injury and microglia morphology as independent variables. Post hoc tests using the FDR method of Benjamini–Hochberg were performed to calculate adjusted *p*-values (q-values) for multiple testing correction. GraphPad Prism software (ver. 10.4.1) was used for statistical analysis and plotting graphs.

## 5. Conclusions

To our knowledge, this is the first study reporting microglia morphology profiles in the cerebral cortex of rats following a mTBI paradigm utilizing a semi-automated and unbiased approach. This proof-of-concept study allows for more granular analysis of microglia states while increasing efficiency and reducing bias associated with the manual determination of morphometrics. This approach has important translational potential as these morphologies are found in both rodent and human brains [13]. In a recent study comparing microglia in the human dorsal anterior cingulate cortex and the mouse cerebral cortex, microglia in both species assumed ramified, hypertrophic, ameboid, and rod-like states, although there were differences in their properties and the distribution of their morphology [36]. Additionally, post-mortem brain tissue [47] as well as the fresh brain biopsy tissue of TBI patients [35] displayed different morphology states, including ramified, hypertrophic, rod-like, and ameboid states. Thus, if this approach can be further optimized for the analysis of microglia in human brains, it could be used to glean valuable information on the pathophysiology and clinical correlates of this debilitating public health problem.

## Figures and Tables

**Table 1:** Comparison of microglial morphological features in sham and CHIMERA groups.

Morphological Feature	Injury Effect	SHAM Mean (SEM)	CHIMERA Mean (SEM)	*p*-Value
**Program: FracLac**				
**Feature Type: Area and Territory Span**				
Foreground pixels	Sham > CHIMERA	7924.03 (279.32)	6586.23 (296.71)	**0.0059**
Maximum span across hull	No difference	237.75 (5.17)	232.58 (6.58)	0.55
Area	No difference	1258.40 (46.94)	1179.67 (61.22)	0.33
Perimeter	No difference	136.09 (2.63)	132.97 (3.75)	0.51
Width of bounding rectangle	No difference	197.84 (4.27)	191.47 (6.89)	0.45
Height of bounding rectangle	No difference	195.37 (4.94)	192.92 (4.23)	0.71
Maximum radius from hull’s center of mass	No difference	256.58 (5.88)	251.13 (6.84)	0.56
Mean radius	No difference	169.81 (3.34)	165.18 (4.62)	0.43
Diameter of bounding circle	No difference	239.73 (5.10)	234.95 (6.66)	0.58
Maximum radius from circle’s center of mass	No difference	119.86 (2.55)	117.48 (3.33)	0.58
Mean radius from circle’s center of mass	No difference	106.27 (2.23)	104.01 (3.00)	0.56
**Feature Type: Cell Shape**				
Density of foreground pixels in hull area	No difference	6.67 (0.32)	6.00 (0.27)	0.14
Span ratio of hull (major/minor axis)	No difference	1.61 (0.032)	1.63 (0.033)	0.63
Circularity	No difference	0.80 (0.0064)	0.79 (0.0063)	0.51
Max/min radii from hull’s center of mass	No difference	5.79 (0.30)	6.04 (0.24)	0.54
Relative variation (CV) in radii from hull’s center of mass	No difference	0.43 (0.010)	0.44 (0.0050)	0.50
Max/min radii from circle’s center of mass	No difference	1.85 (0.031)	1.84 (0.042)	0.90
Relative variation (CV) in radii from circle’s center of mass	No difference	0.15 (0.0034)	0.15 (0.0054)	0.57
**Program: Skeleton Analysis**		**SHAM**	**CHIMERA**	
**Feature Type: Branching Complexity**				
Number of branches	No difference	77.85 (2.05)	70.12 (3.08)	0.059
Number of junctions	Sham > CHIMERA	41.86 (1.14)	37.34 (1.71)	**0.048**
Number of end point voxels	No difference	27.39 (0.69)	25.71 (0.96)	0.179
Number of junction voxels	Sham > CHIMERA	83.49 (2.33)	74.25 (3.25)	**0.039**
Number of slab voxels	No difference	973.95 (20.56)	890.69 (36.15)	0.071
Number of triple points	Sham > CHIMERA	39.22 (1.04)	34.91 (1.61)	**0.045**
Number of quadruple points	No difference	2.55 (0.16)	2.35 (0.12)	0.32
**Feature Type: Branch Length**				
Maximum branch length	No difference	14.68 (0.34)	15.49 (0.59)	0.26
Average branch length	No difference	3.73 (0.065)	3.81 (0.094)	0.46

## Data Availability

Data is contained within the article: The original contributions presented in this study are included in the article. Further inquiries can be directed to the corresponding author.

## Glossary

**Shape terminology**	
Hull	the smallest convex shape that completely encloses the cell.
Endpoint	The terminus or end of a microglial process or branch.
Junction	The point at which the processes of a microglial cell divide or connect.
Slab voxels	The segment of a microglial process between an endpoint and junction.
**Feature terminology**	
Foreground pixels	The pixels representing the cell after thresholding.
Maximum span across hull	The longest distance between any two points on the convex hull of the cell.
Area	The number of pixels that make up the cell.
Perimeter	The length of the cell’s outer boundary.
Width of bounding rectangle	The horizontal extent of the smallest rectangle that encloses the cell.
Height of bounding rectangle	The vertical extent of the smallest rectangle that encloses the cell.
Maximum radius from hull’s center of mass	The longest distance from the convex hull’s centroid (center of mass) to its outer boundary.
Mean radius	The average distance from the cell’s center to its outer boundary.
Diameter of bounding circle	The diameter of the smallest circle that completely encloses the cell.
Maximum radius from circle’s center of mass	The longest distance from centroid to the cell’s boundary.
Mean radius from circle’s center of mass	The average distance from the cell’s centroid to any pixel of the cell.
Density of foreground pixels in hull area	The fraction of the convex hull that is filled by the cell’s pixels
Span ratio of hull (major/minor axis)	The ratio of the convex hull’s longest axis to its shortest axis.
Circularity	How circular the cell is on a scale of 0 to 1.0, where 1.0 is a perfect circle.
Max/min radii from hull’s center of mass	The ratio between the longest and shortest distances from the convex hull’s centroid to its edge.
Relative variation (CV) in radii from hull’s center of mass	The coefficient of variation of the distances between the convex hull’s centroid and outer boundary.
Max/min radii from circle’s center of mass	The ratio between the longest and shortest distances from the bounding circle’s centroid to the cell’s outer boundary.
Relative variation (CV) in radii from circle’s center of mass	The coefficient of variation of the distances between the bounding circle’s centroid and outer boundary.
# of branches	The quantity of branch segments in the skeletonized structure.
# of junctions	The quantity of points where two or more branches converge in the skeleton.
# of end point voxels	The quantity of voxels at the ends of branches in the skeleton.
# of junction voxels	The quantity of voxels that are part of a junction.
# of slab voxels	The quantity of voxels that form the segments between junctions and endpoints in the skeleton.
# of triple points	The quantity of junctions in the skeleton where exactly three branches meet.
# of quadruple points	The quantity of junctions in the skeleton where exactly four branches meet.
Maximum branch length	The length of the longest continuous branch in the skeleton.
Average branch length	The mean length of all branches in the skeleton.
